# Resistance of *Varroa destructor* against Oxalic Acid Treatment—A Systematic Review

**DOI:** 10.3390/vetsci11090393

**Published:** 2024-08-26

**Authors:** Yvonne Kosch, Christoph Mülling, Ilka U. Emmerich

**Affiliations:** 1 Institute of Veterinary Anatomy, Histology and Embryology, Faculty of Veterinary Medicine, Leipzig University, An den Tierkliniken 43, 04103 Leipzig, Germany; gw32ewuk@studserv.uni-leipzig.de (Y.K.);; 2 Institute of Pharmacology, Pharmacy and Toxicology, Faculty of Veterinary Medicine, Leipzig University, An den Tierkliniken 15, 04103 Leipzig, Germany

**Keywords:** *Varroa destructor*, *Apis mellifera*, resistance, oxalic acid

## Abstract

**Simple Summary:**

Economically, Varroa destructor is the most important parasite to honey bees. There are many ways to deal with it, including pharmaceutical and biotechnological treatments. However, the mite has become resistant to many synthetic pesticides. There is little research on its response to organic acids. This report examines the question of whether it could become resistant to oxalic acid. The review uses literature from the past 30 years, and calculates and reviews the annual median efficacy for different application methods. If an efficacy of 70% or more is achieved, it can be concluded that the organism is not resistant. There is no evidence of resistance development, despite some outliers, which can be explained by the studies. Further tests are required to confirm the results.

**Abstract:**

As *Varroa destructor* is one of the most important pathogens of *Apis mellifera*, there are numerous treatment methods, including pharmaceutical and biotechnological approaches. However, the rapid development of resistance to synthetic acaricides by *Varroa destructor* has become a significant concern. To date, there have been no investigations into the development of resistance to organic acids. This review examines the potential risk of oxalic acid resistance development by evaluating literature sources from the past 30 years following the PRISMA 2020 guidelines. Median annual efficacies are calculated and reviewed over time for several application methods. An efficacy higher than 70% is determined as not resistant. Independent of the method of application, no resistance development can be observed, although there are some outliers of the annual median. These outliers can be explained by brood status or study setting. However, the result is limited by the low number of efficacy values, and further standardised studies are needed.

## 1. Introduction

*Varroa destructor* is considered to be one of the most significant parasites of *Apis mellifera*. The first parasitisation of *Apis mellifera* with *Varroa destructor* was described in Hong Kong in 1962 [1]. It was first reported in Germany in 1977 [2] and has now spread to bee colonies around the world. Australia stopped eradicating *Varroa destructor* in September of 2023 [3]. This rapid global spread highlights the importance of protecting *Apis mellifera* and implementing effective treatment for *Varroa destructor*. The control of *Varroa destructor* is a primary goal of veterinary medicine, as honey bees are a key pollinator species for global food production [4]. Various control methods, such as pharmacotherapeutic treatment with synthetic acaricides and organic acids as well as biotechnological control methods are available. *Varroa destructor* has already developed resistance to several pharmacotherapeutic treatment options. Around 30 years after the first parasitisation of *Apis mellifera*, *Varroa destructor* developed resistance to tau-fluvalinate and amitraz in 1991, both of which had an initial efficacy of 90 % (reviewed by [5]). Resistance to flumethrin and coumaphos were reported in 1995 and 2001, respectively [5]. The “drug resistance” of a parasite is defined as the reduction in effectiveness of a medication in an originally susceptible population. It reduces or eliminates the effectiveness of antiparasitic drugs in controlling parasites [6]. Such resistance can be diagnosed by analysing the reduction in the pathogen in a selected population after treatment with the tested compound and determining efficacy [6]. The European Medicines Agency (EMA) published the *Guideline on veterinary medicinal products controlling Varroa destructor parasitosis in bees* in 2021. A revision is in progress, and the respective concept paper will be finalised in the fourth quarter of 2024. The aim of this guideline is to provide guidance on the efficacy and safety determination of varroacides [7]. The efficacy of the medicinal products controlling *Varroa destructor* is affected by the application method, the number and interval of treatment, the product dose, the hives’ brood status, and the environmental conditions [7].

The synthetics flumethrin and amitraz are authorised for the treatment of honey bees against *Varroa destructor* in Germany [8]. Pyrethroids are only available in pharmacies, and amitraz is only available by prescription in Germany [8]. Due to the high level of resistance to synthetic acaricides, effective alternatives are needed. Organic acids are one of the substance classes commonly used instead of or in combination with synthetic varroacides [9]. In the USA, the use of oxalic acid has been increasing since 2013 [10]. In Germany, oxalic acid, formic acid, and lactic acid are approved as varroacides [8]. Veterinary drugs with these organic acids are freely available today [8]. Oxalic acid is not known to have a negative effect on honey bee health, hive development, or behaviour [11,12,13,14,15,16,17,18,19]. Oxalic acid is a natural compound in honey [20] and there is a low risk of accumulation in wax and honey [13,21,22]. There is no known resistance of *Varroa destructor* to oxalic acid to date [23]. As resistance of *Varroa destructor* to tau-fluvalinate, amitraz, flumethrin, and coumaphos has developed so rapidly, possibly due to the overuse of these substances and with an increasing use of organic acids, the potential risk of developing resistance to these organic acids needs to be investigated.

The aim of this review is to examine the risk of potential resistance development in *Varroa destructor* to treatment with organic acids under field conditions depending on the method of application, organic acid, and the type of hive. The results for oxalic acid are reported in this article. Formic acid and lactic acid will be considered in a future publication.

## 2. Materials and Methods

This systematic literature review follows the PRISMA 2020 guidelines [24]. The selected literature sources were obtained from the medical database PubMed^®^ and the multidisciplinary database Web of Science^TM^. The literature collection for this study also comprises relevant publications found in the library catalogues of the German National Library and the University of Leipzig.

**Figure 1:**
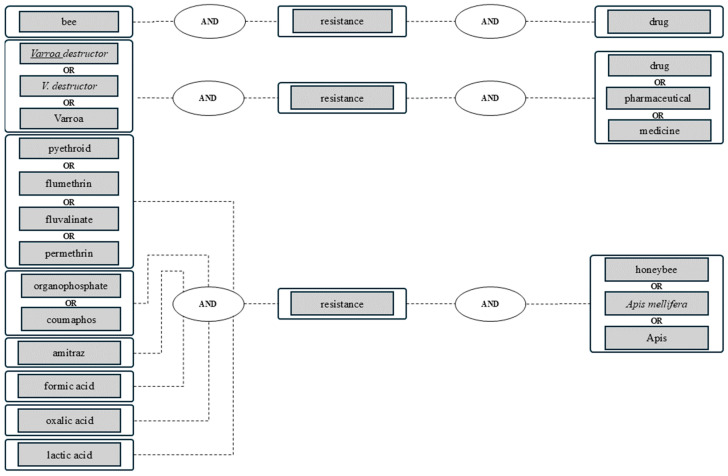
Search terms and combination examples, linked with Boolean operators (bold printed); search and alert period: 1 January 2023 to 31 December 2023; searched databases: PubMed^®^, Web of Science^TM^, German National Library, Library of the University of Leipzig.

In order to obtain a complete and comprehensive result, several synonyms were selected for the full-text search. Search terms were linked with Boolean operators (AND, OR) (Figure 1).

The abstracts of the resulting publications were screened for inclusion criteria. The topic must be related to *Varroa destructor* resistance to organic acids, alternative treatments, reasons for resistance, effective treatment against *Varroa destructor*, organic acids as a comparative treatment or control treatment, mechanisms of toxicity, and resistance development or negative effects of organic acids on honeybees. After checking the abstract for the inclusion criteria, duplicates were removed. The selected literature sources were reviewed for relevant information to determine the potential development of resistance in *Varroa destructor* to oxalic acid, removing all references lacking relevant information. The review was performed by checking for comparable parameters and documenting them in a Microsoft Excel spreadsheet. These parameters included the year, location and type of study, the study’s main objective, the mite monitoring method, the control treatment, the hive constitution, the climatic conditions, the treatment material and method of application, and the mean efficacy of the different treatments. Essential information in relation to the mite monitoring was the type and number of mite counts and the intervals. Hive constitution is defined as the type and number of hives and brood status. Treatment details such as number and interval, method of application, and dose were required. The screening of the publications was carried out by only one reviewer instead of three or more, as recommended by the PRISMA guidelines. However, in the context of examining decreasing efficacy over time, the potential for bias caused by a single reviewer’s subjective view is not a concern because any potential bias would affect efficacy values equally over time, leaving the general efficacy tendencies over time unaffected. The geographic information system Quantum (Qgis) [25] was used to map the location of the studies.

### Data Processing

The collected data needed to be processed for analysis. The doses used in the literature were different and not directly comparable. In order to make different hive sizes more comparable, doses over all treatments were normalised relative to comb area for trickling and spraying (Equation (1)) and relative to hive volume for vaporisation and exchange via direct contact (Equation (2)). Where possible, the given units, such as grams of oxalic acid or oxalic acid dihydrate per litre of treatment solution and number and size of frames or hives, were converted to the target units, as shown in Equations (1) and (2). Where conversion was not possible due to missing values, the original unit was retained. Sometimes, it is not clear whether the values given refer to oxalic acid or oxalic acid dihydrate. If not clearly defined, it was assumed that oxalic acid dihydrate was used, because it is the most commonly available substance for treatment. The efficacy values found in the literature and expressed as percentages were widely scattered and the number of values for each application method for the individual years was small. Therefore, an annual median was calculated.

(1) $$D_{A}=\frac{v\times c}{a\times b}$$

The calculation method for trickling and spraying, $D_{A}$: areal dose as target dose for trickling and spraying; v: volume of oxalic acid dihydrate solution per frame; c: concentration (mass/volume); a: frame width (bottom beam measured); b: frame height.

(2) $$D_{V}=\frac{m\times n}{l\times w\times h}$$

The calculation method for vaporisation and exchange via direct contact, $D_{V}$: volume dose of hive volume as target dose for exchange via direct contact and vaporisation; l: length of hive; w: width of hive; h: height of hive; m: mass of oxalic acid dihydrate; n: number of used blocks or strips.

The normalised doses were divided into different dose ranges and an annual average dose was calculated for the analysis of dose changes over the years. The average value was chosen instead of the median because the dosages have a lower number of outliers compared to the efficacy. The dose ranges of the categories were determined differently due to varying amount of values and large differences in the dose level depending on the application method.

## 3. Results

**Figure 2:**
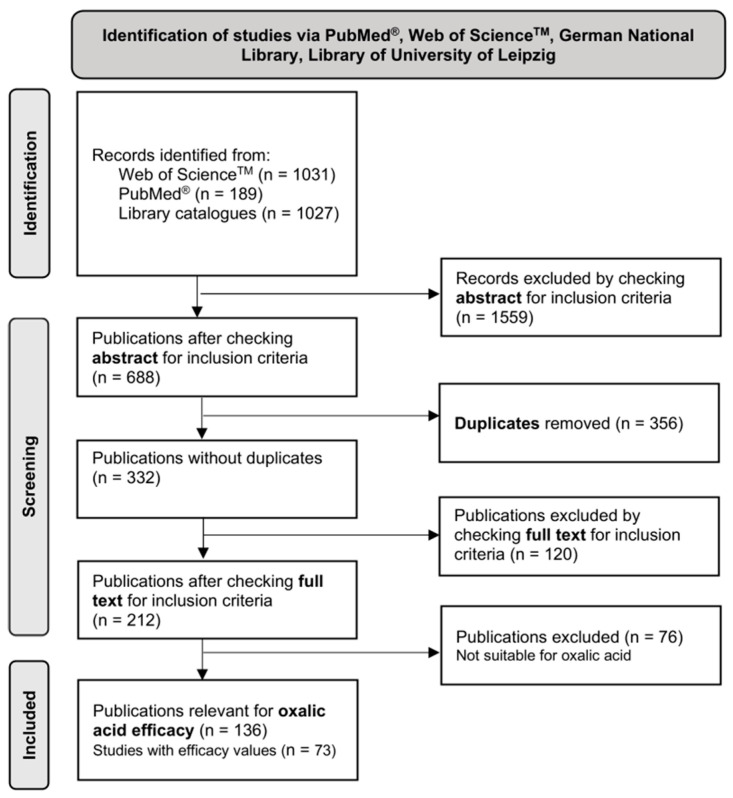
Flowchart of literature search strategy according to PRISMA 2020 guidelines [24].

Initially, 2247 reference sources were found in the Web of Science^TM^ database (*n* = 1031), PubMed^®^ (*n* = 189), and the two library catalogues (*n* = 1027). After checking the abstracts for inclusion criteria, 688 sources remained in total. The remaining literature sources without duplicates resulted in 332 publications from Web of Science^TM^ (*n* = 289), PubMed^®^ (*n* = 37), and the libraries (*n* = 6). Upon full-text review, 212 sources were found to be relevant to the study question, with 136 being suitable for the observations on oxalic acid treatment (Figure 2). Some of them did not include efficacy values, but did include relevant information on oxalic acid efficacy discussion in general. This information relates, for example, to mite decline over time under laboratory conditions without determining efficacy [26] and the mode of action of oxalic acid or possible resistance mechanisms of Varroa mites [27,28,29,30,31]. Only the literature sources that included efficacy values or substantial background information for the discussion in this publication were finally used and are mentioned in the “References” section.

**Figure 3:**
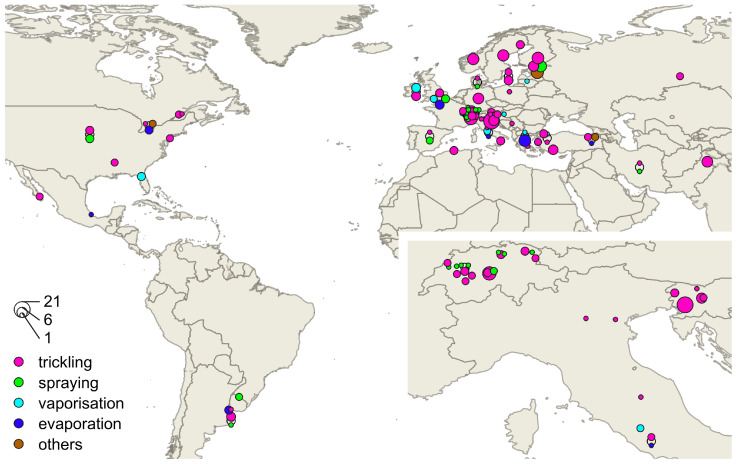
Geographic distribution of oxalic acid studies containing a location at least at country level. Symbol size represents the number of studies at the same location (generated with QGIS [25]).

The range of 274 individual efficacy values of oxalic acid in 73 literature sources varied from 5% [32] to 100% [33,34] between 1994 [35,36] and 2022 [37]. Some of these 73 sources included more than one study setting and therefore reported multiple efficacy values. A total of 224 (81.8%) values were higher than or equal to 70% efficacy, and 146 values (53.3%) were higher than or equal to 90% efficacy. The annual medians for all application methods resulted in only three years below 70% efficacy and 16 years below 90% efficacy. The data included 32 countries from around the globe (Figure 3).

The literature sources described various application methods, which were grouped into seven categories for comparison purposes: trickling (*n* = 188), spraying (*n* = 31), vaporisation (*n* = 26), exchange via direct contact (*n* = 18), dusting (*n* = 3), and others (*n* = 8). The “others” category consists of application in bee cake under field conditions [38], methods under laboratory conditions [39], and undefined methods [20,40]. In addition, 146 values came from broodless colonies, while 102 came from hives with broods. The remaining 26 values were from hives with undefined brood status. The number of treatments varied and sometimes were not mentioned, but most of the efficacy values resulted from one treatment.

### 3.1. Efficacy Measurement

The methods of mite monitoring and efficacy measurement differed between the individual studies. There are three different kinds of mite counting: washing, bottom board sampling, and brood cell opening. These methods were used individually or in combination with each other. The number of counts ranged from 2 [41,42] to 15 [42] times with intervals of 1 [37,43,44,45,46,47] to 15 [48] days.

The “washing method” describes the harvesting of 100 [49] to 200 [50] bees and detaching the mites by shaking [37,49,50,51,52], rolling [53], flotation [38,41,54], or waterjetting [48]. The solvents used in the washing method ranged from ethanol [19,49,50,51,53,55,56,57,58] over water with [52,54] or without detergent [48] and unknown liquid [41] to powdered sugar [59]. After washing the bees, the mites were counted to determine the infestation.

“Bottom board sampling” means counting fallen mites before or after treatment on the bottom board of the hive. To guarantee the most comprehensive count and to avoid reinfestation, some studies used protective nets [60] or sticky bottom boards [1,15,16,17,18,19,22,32,33,34,38,42,43,44,45,47,51,55,56,57,61,62,63,64,65,66,67,68,69,70,71,72,73,74,75,76,77,78,79,80,81,82,83,84,85].

Determining the mite infestation level by opening 200 to 300 brood cells per hive was used several times [19,38,49,50,54,56,64]. In this approach, drone brood cells are opened and the content is examined for mites.

The effectiveness of a treatment can also be calculated in different ways. Some publications did not describe the calculation method exactly, but most of the studies divided the counted mites during the tested treatment by the total mite number, counted during the control treatment with another substance and the tested substance [14,22,36,38,40,43,44,47,63,66,70,75,77,81,83,84,85,86,87] or calculated as a percentage of infested brood cells or mite number on bees after treatment compared to the initial number of infested brood cells or mite number on bees [46,59,64,76,82,88]. Overall, 89% of the efficacy values were determined by counting mites by bottom board sampling and dividing the number of counted mites during the tested treatment by the total mite number. These efficacy values varied from 5 to 100%.

### 3.2. Trickling

The application method of “trickling” is performed by dribbling a specific volume of oxalic acid in water or in a water–sugar solution onto the bees between the frames [87]. The used volumes per colony vary from 30 millilitres [89] to 100 millilitres [67] of oxalic acid solution. A volume between 3 millilitres [90] and 25 millilitres of oxalic acid solution per frame space [67,68,85] is applied. The normalised target unit for the trickling method is milligrams of oxalic acid dihydrate per square decimetre of frame surface. For example, using Equation (1), six millilitres of a four per cent oxalic acid dihydrate solution per frame of a Dadant Blatt hive result in an areal dose $D_{A}$ of

$$\frac{6\;mL\times 40\;mg/mL}{4.35\;dm\times 3.0\;dm}\approx 18.4\;mg/{dm}^{2}$$

**Figure 4:**
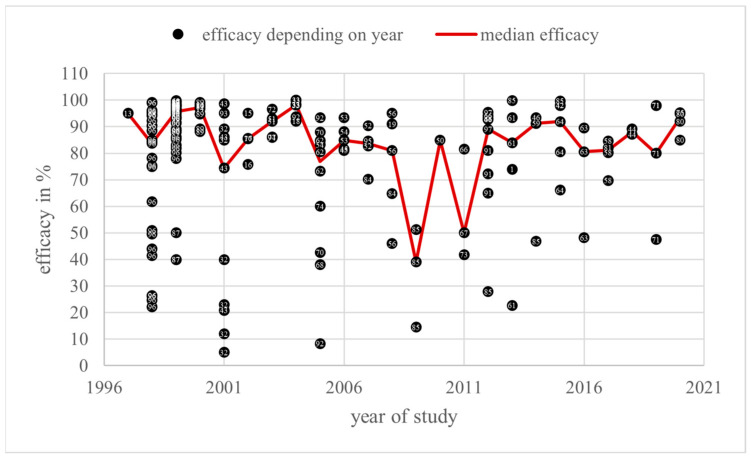
Efficacy values and annual median efficacy of “trickling”; References [1,13,14,15,16,18,19,32,33,41,42,43,44,45,46,50,51,52,53,54,56,58,59,60,61,62,63,64,66,67,68,70,71,72,73,74,75,77,80,81,84,85,87,89,91,92,93,94,95,96,97] correspond to the numbered data points.

A total of 185 efficacy values in percent and three in other units were found for the category “trickling” in a total of 55 literature sources overall. Most of the values come from studies from 1999, with 27% of the 185 values for this category (*n* = 50). The lowest numbers were found for 1997 and 2010, respectively (*n* = 1). Efficacy varied from 5 [32] to 100% [33]. A total of 149 (80.5%) values were higher than 70% in the period from 1997 to 2020. Moreover, 91 (49.2%) values were higher than 90% over the period from 1997 to 2020. As a result, there were 36 tests (19.5%) with efficacy lower than 70%. Out of the 24 years’ worth of studies, only two annual medians of efficacy, in 2009 and 2011, were lower than 70% (Figure 4).

A total of 96 of the values for “trickling” came from the treatment of broodless hives, and 73 came from bee colonies with broods. The remaining 16 had an unknown brood status. Different doses of oxalic acid were used, and can be divided into the seven areal dose ranges $D_{A}$ shown in Table 1. 

T_1_ comprises data over a period from 1998 to 2013, T_2_ from 1998 to 2019, T_3_ from 1997 to 2017, T_4_ from 2001 to 2020, T_5_ from 2005 to 2020, T_6_ from 2004 to 2020, and T_un_ from 1998 to 2020. The areal dose $D_{A}$ in group T_un_ is not calculable because of the laboratory conditions, where no hive or frame was used [85], or the hive type was not given [19,41,44,45,50,53,59,63,71,74,75,90,95,97] and therefore a surface size is not known. In some literature sources of the T_un_ group, the volume of trickled solution was not mentioned [43,70,72,93], so an areal dose is not calculable. The used solution concentrations ranged from 2.5% [63] to 7.5% [19,44] oxalic acid dihydrate. A volume of 3 [90] to 10 [85] millilitres per frame was used in this group.

**Figure 5:**
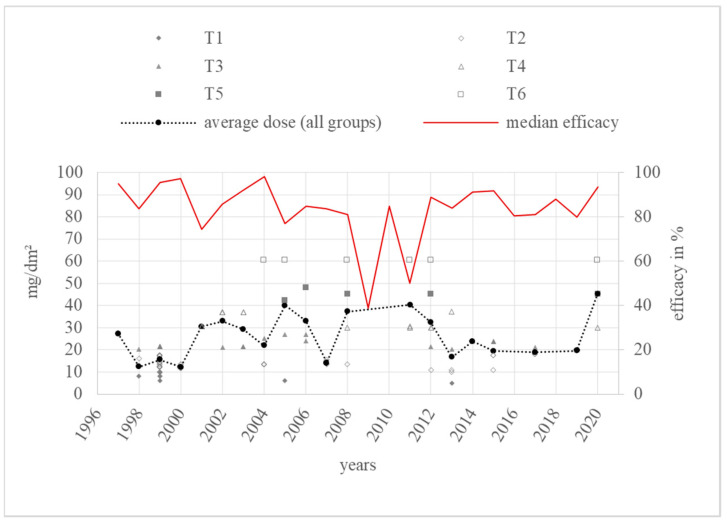
Doses and annual average doses of “trickling”.

The dose modifications over the years and the annual average doses are shown in Figure 5.

The annual average dose over all groups in the category “trickling” ranged from 12.3 mg/dm^2^ (T_2_) to 45.2 mg/dm^2^ (T_5_). The highest dose group, T_6_, was used in only one or a maximum of two trials per year in 6 years over a period of 16 years. Most doses relate to group T_2_. In total, six substantial dose increases were observed in 2001, 2005, 2008, and 2020. The highest annual average dose was used in 2020, consisting of three different doses from group T_4_ [80], T_5_ [80], and T_6_ [80].

### 3.3. Spraying

The application method “spraying” is described as the manual spraying of an oxalic acid dihydrate in water solution onto the surface of the frames occupied by bees using an atomiser [35]. The volume and concentration of the used solution and the used atomisers varied in the different study settings. Aligned with “trickling”, the doses for the “spraying” category were normalised to milligrams of oxalic acid dihydrate per square decimetre of frame surface using Equation (1).

**Figure 6:**
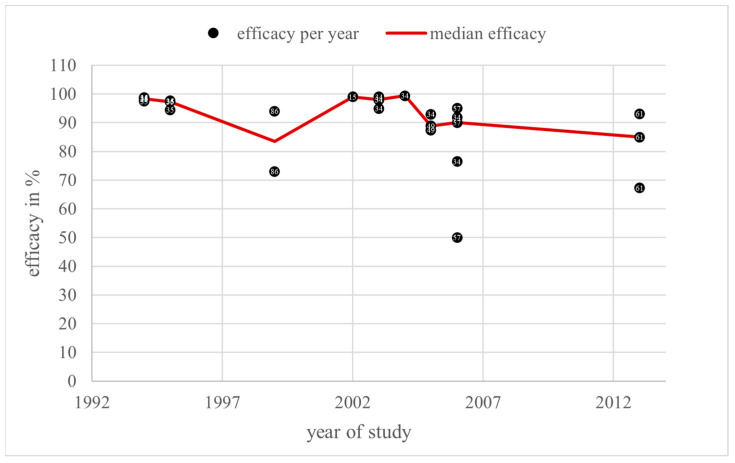
Efficacy values and annual median efficacy of “spraying”; References [15,34,35,36,49,57,61,86] correspond to the numbered data points.

Overall, 29 efficacy values were found in the category “spraying” in a total of 10 literature sources from the years 1994 to 2013. The efficacy values varied from 50% [57] to 99.4% [34]. Here, 27 of the total 29 efficacy values (93.1%) were higher than or equal to 70%, and the remaining 2 (6.9%) lower ones were found in 2006 and 2013. The annual median efficacy values were always higher than 70%. Only in 1999, 2005, and 2013 were the median efficacy values lower than 90% (Figure 6).

The brood status varied between the reported efficacy values. In total, 7 were measured in colonies with broods and 22 in broodless hives. As shown in Table 2, five dose groups were formed.

S_1_ and S_2_ comprise data over the period from 1994 to 2013, and S_3_ from 2002 to 2013. The single value in group S_4_ was measured in 2003. For S_un_, an areal dosage was not calculable because the hive type was not given in the study from 1998 [90], or the bees were treated in packages after transport and not in the original frame in 2006 [57]. Here, 1.1 to 3.4 millilitres per bee package of a 3.5% oxalic acid dihydrate solution [57] or 3 to 4 millilitres of a 3.0% oxalic acid dihydrate solution per frame [90] was used.

**Figure 7:**
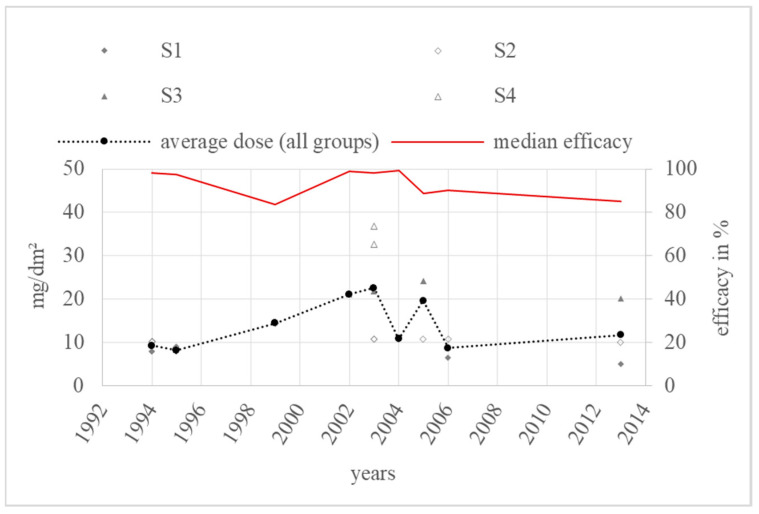
Doses and annual average doses of “spraying”.

The dose modifications over the years and the annual average doses are shown in Figure 7. 

The annual average dose over all groups in the category “spraying” ranged from 8.3 mg/dm^2^ (S_1_) in 1995 to 22.6 mg/dm^2^ (S_3_) in 2003. The highest annual average dose consisted of two values from S_2_ [34] and S_4_ [34,78], and one value from group S_3_ [34]. The highest dose group, S_4_, was used in two trials in 2003, but only once was an efficacy value in percentage given. The most used dose group was S_2_. Overall, the annual average dose rose sharply once from the year 1994 to the period 1999 to 2003. After that, the average dose decreased from 19.7 mg/dm^2^ in 2005 to 11.8 mg/dm^2^ in 2013, therefore remaining stable in dose group S_2_.

### 3.4. Exchange via Direct Contact

The application method of “exchange via direct contact” is described as the impregnation of different materials with oxalic acid dihydrate and subsequent transfer from these materials to the bees via direct contact to the carrier material and exchange among the bees. The materials used ranged from cellulose strips [22,45,47] over vermiculite blocks [67,68] to soaked towels [83]. Oxalic acid dihydrate was dissolved in various solvents. The liquids varied, including water [45,83], sugar water [82], ethanol [67,68], and glycerine [47,83]. The target unit in this category is milligrams of oxalic acid dihydrate per litre of hive volume. Therefore, if, e.g., AluenCap^®^ with four strips of 10,000 milligrams of oxalic acid dihydrate is used in a Dadant Blatt hive, according to Equation (2), a volume dose of approximately

$$\frac{10000\;mg\times 4}{4.35\;dm\times 4.35\;dm\times 3.33\;dm}\approx 635\;mg/L$$

is administered to the bees.

**Figure 8:**
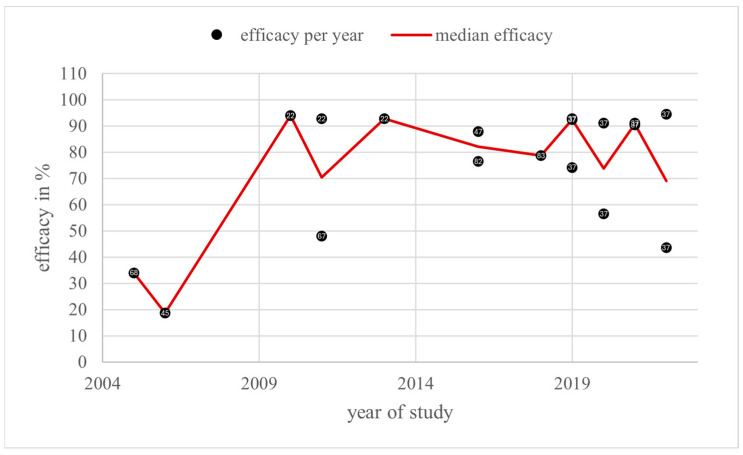
Efficacy values and annual median efficacy of “exchange via direct contact”; References [22,37,45,47,67,68,82,83] correspond to the numbered data points.

The category “exchange via direct contact” includes a total of 18 efficacy values from 2005 to 2022 from eight literature sources. The efficacy of these studies ranged from 18.7% [45] to 94.5% [37]. Moreover, 72.2% of the 18 values were higher than or equal to 70% efficacy. Only three annual medians of efficacy, for 2005, 2006, and 2022, were lower than 70% (Figure 8).

Six values came from colonies with broods, and two from broodless hives. For ten values, no information on the brood status was provided. Dose groups were created as shown in Table 3.

One efficacy value in the E_un_ group had no described dose. The doses for a further five values were described in grams of oxalic acid dihydrate per colony, but the exact hive volume or type was not specified. In addition, 40,000 milligrams of oxalic acid dihydrate per colony in an unknown hive volume led to an efficacy range of 87.8% [47] to 94% [22], while 10,000 milligrams resulted in an efficacy of 92.8% [22]. The values for this group were collected from 2006 to 2022, for E_1_ were from 2005 to 2020, and for E_2_ were from 2019 to 2022.

**Figure 9:**
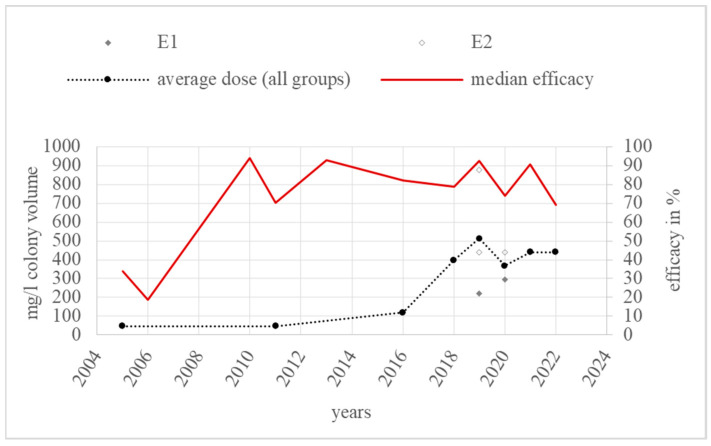
Doses and annual average doses of “exchange via direct contact”.

The dose modifications over the years and the annual average doses are shown in Figure 9.

The annual average dose over all groups in the category “exchange via direct contact” ranged from 47.6 mg/L (E_1_) to 440.5 mg/L (E_2_). The highest annual average dose in the year 2019 consisted of one dose value from group E_1_ [37] and two values from E_2_ [37]. The highest dose group, E_2_, was used six times from 2019 to 2022, and E_1_ was also used six times from 2005 to 2020. Overall, two dose increases can be observed from 2011 to 2016 and from 2016 to 2018. From 2005 to 2022, the annual average doses rose from E_1_ to E_2_.

### 3.5. Vaporisation

“Vaporisation” means the evaporation of oxalic acid dihydrate crystals using additional heating by a commercial vaporiser like VARROX^®^ [17,48,55,59,63,79,98] or Sublimox^®^ [76]. Like in the category “exchange via direct contact”, the target unit is milligrams of oxalic acid dihydrate per litre of hive volume. For example, the application of 2000 milligrams of oxalic acid dihydrate to a colony housed in a Dadant Blatt hive results in a volume dose of about

$$\frac{2000\;mg}{4.35\;dm\times 4.35\;dm\times 3.33\;dm}\approx 31.7\;mg/L$$

**Figure 10:**
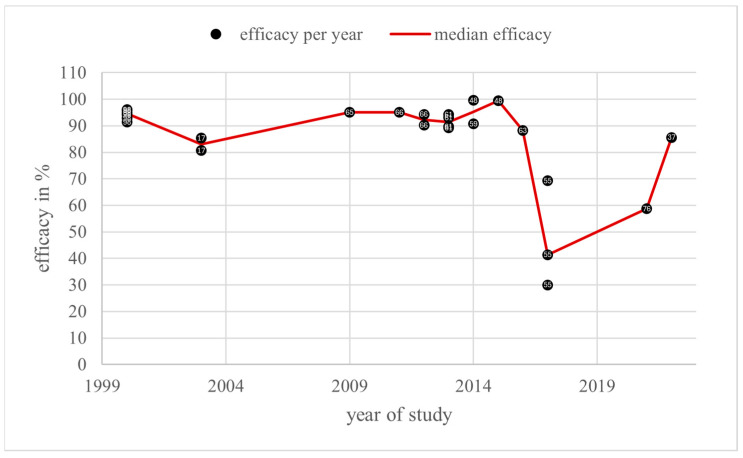
Efficacy values and annual median efficacy of “vaporisation”; References [17,37,48,55,59,61,63,65,66,76,98] correspond to the numbered data points.

In total, 25 efficacy values from 2000 to 2022 were found in the category “vaporisation” in 12 literature sources. The efficacy varied from 30.0 [55] to 99.6% [48]. In addition, 21 (84%) of these values were higher than or equal to 70%, and 60% of the values were higher than or equal to 90%. Only 2 of 22 years had a median efficacy lower than 70% (Figure 10).

The tested colonies were of different brood statuses. Eight values came from colonies with broods, thirteen from broodless colonies, and four from colonies with unknown brood status. Four different dose groups were formed, as shown in Table 4.

Group V_1_ comprised data over a period from 2003 to 2017, V_2_ from 2003 to 2022, V_3_ from 2011 to 2017, and V_un_ from 2000 to 2021. Group V_un_ consisted of nine values varying from 58.7% [76] to 96% [98]. The volumes of colonies tested in this group were not given. The doses used were given in grams of oxalic acid dihydrate per colony. Moreover, 1.4 g per colony led to an efficacy range from 92.9% [98] to 95.7% [98], while 2.0 g per colony reached an efficacy range from 58.7% [76] to 88.3% [63]. The treatment with 2.8 g oxalic acid dihydrate per colony achieved an efficacy range from 91.4% [98] to 96% [98], while a dose of 3.6 g per colony resulted in an efficacy of 90.8% [59].

**Figure 11:**
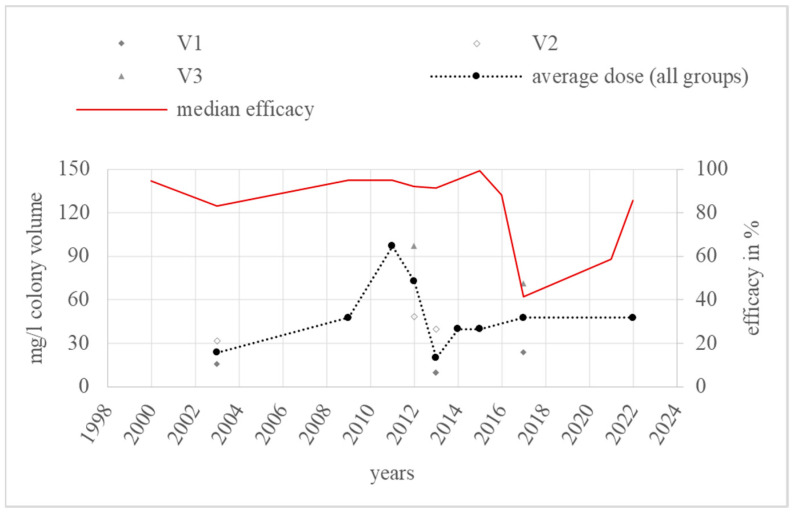
Doses and annual average doses of “vaporisation”.

The dose modifications over the years and the annual average doses are shown on the right in Figure 11. 

The annual average dose over all groups in the category “vaporisation” ranged from 19.9 mg/L (V_1_) in 2013 to 97.1 mg/L (V_3_). The highest dose group, V_3_, was used three times [55,66]. Most doses related to group V_2_ [17,37,48,55,61,65,66]. Overall, one significant dose increase can be observed from 2003 to 2011. The annual average dose decreased sharply in 2013. The highest annual average dose was used in 2011, consisting of only one dose of group V_3_.

### 3.6. Dusting

The dusting method is described as producing a powder of oxalic acid dihydrate and sugar and administering via a newspaper sheet [67,68,83].

Only three values from the years 2005, 2011, and 2018 belonged to the category “dusting” and originated from three literature sources. The efficacy in this category varied from 28% [68] to 53% [83].

### 3.7. Others

The “others” category consists of 14 values from the years 2005 to 2013, obtained from three literature sources. The efficacy under laboratory conditions (*n* = 13) varied from 10% [39] to 100% [34]. Only one value originated from a treatment under field conditions. The oxalic acid was applied with bee cake and led to an efficacy of 66.7% [38].

## 4. Discussion

The aim of this literature review was to check published data for first indications for the possible development of resistance in the mite *Varroa destructor* to organic acids such as oxalic acid.

The *Guideline on veterinary medicinal products controlling Varroa destructor parasitosis in bees*, published by the European Medicines Agency (EMA), defines the efficacy of a varroacide as the mite mortality rate in percentage, calculated by dividing the number of fallen mites after the tested treatment by the total number of fallen mites after the tested treatment and critical test, multiplied by 100 [7]. These efficacy values, collected from the literature sources of this review, are shown in Figure 4, Figure 6, Figure 8 and Figure 10 depending on the year of study. The EMA prefers an efficacy result of more than 90% for non-synthetic substances to reduce the risk of resistance development [7]. For efficacy determination, a standardised trial protocol should be used, where the fallen mites are counted using the bottom board sampling method. A fitted mesh tray should be preferred. The counting of dead mites should take place pre- and post-treatment. A follow up treatment, called a “critical test” [7], must be applied, using an unrelated product with a documented efficacy of 95%. The document also points out that a medicinal product for varroa control should always be part of an integrated pest management (IPM) system [7]. Therefore, the use of oxalic acid as a medicinal product for the control of *Varroa destructor* should be combined with another form of pest management to guarantee an efficacy of at least 90%, although oxalic acid alone can also achieve this efficacy under ideal conditions. With this in mind, and the fact this review only considers treatments where oxalic acid is the sole active substance used under a wide range of different conditions, an efficacy of at least 70% for an application of oxalic acid, without additional active substances or control methods, is defined as effective and not resistant in this discussion.

But not only the efficacy value itself should be examined. The dose changes over the years can also be an indication of resistance development. If the dose increases while the efficacy remains the same, the resistance of the target could be covered by the higher dose. The target is eliminated effectively because of a higher dose and not because of an effective drug. Therefore, resistance masked by dose escalation must be ruled out.

To answer the initial question of resistance development, the efficacy of oxalic acid over the years was evaluated. The application method, drug dose, number of treatments, brood status, and study setting were considered according to the EMA recommendations for efficacy studies. Furthermore, dose escalations combined with a stable efficacy level or dose adaptation immediately after lower efficacy levels over the years were discussed. The categories “dusting” and “others” were not analysed due to insufficient data.

### 4.1. Efficacy Measurement

Most of the given efficacy values were determined by bottom board sampling in combination with calculating the efficacy by dividing the number of fallen mites after treatment by the total number of fallen mites, including those killed by the critical test. As bottom board sampling causes 89% of the efficacy values and is recommended by the EMA for mite counting [7], the methodology employed for efficacy measurement is not the focus of this discussion. But, if necessary, it is considered in the discussion of efficacy outliers of the different application methods. Overall, there is a wide range of efficacy measurement methods, which leads to limited comparability. Therefore, standardised tests are required to make reliable statements about resistance development.

### 4.2. Trickling

In 24 years of trickling studies from 1997 to 2020, there were only two years with an efficacy median below 70%. In as many as eight of these years, most notably in the final year, 2020, the efficacy was higher than the 90% requested by the EMA.

The median efficacy in 2009 was 39%, derived from a single study with values of 51.3%, 14.5%, and 39% [85]. This study was carried out under laboratory conditions. All efficacy values, even from other years, derived from this study were less than or equal to 51.3% [85]. The other reviewed sources in this category were performed under field conditions; consequently, a direct comparison would not be meaningful and those values can be eliminated.

The second notable year is 2011. The median of 50% was the result of three values from three different sources with a particularly wide spread: 41.8%, 50%, and 81.5%. All of them were obtained from field trials with brood-positive hives [66,67,73]. The mite sampling was performed by counting fallen mites on sticky bottom boards in every setting according to the recommendation of the EMA. Other variables that may give rise to different efficacies include control treatment, oxalic acid dose, and differences in the amount of broods. For the control treatment, Perizin^®^ [67] and Checkmite+^®^ [66,73] were used. However, both contain the organophosphate coumaphos as their active ingredient, which eliminates differences in the critical test as a major factor for the wide spread of the calculated oxalic acid efficacies. If different oxalic acid doses caused the differences between the test results, a higher efficacy would be expected for a higher dose. However, the doses did not correlate with the efficacy results: out of the dose groups used, i.e., T_3_, T_4_, and T_6_, T_6_, as the highest dosage, did not lead to the highest efficacy of 81.5%, but instead to an efficacy of 50%. Therefore, the dose does not explain the poor outcomes of 41.8% and 50% and cannot be the reason for the efficacy variations in 2011. Looking more closely at the brood status, Coffey et al. described the status of their colonies as having little brood area. Due to the climatic and geographic differences, their colonies in December in Ireland [66] will very likely have contained barely any covered brood, whereas for both Emsen et al. in early autumn in Erzurum in Turkey [67] and Gregorc et al. in August and September in Slovenia [73], the unquantified amount of brood can be expected to contain significant covered brood areas. Considering that oxalic acid is less effective in controlling *Varroa destructor* in the presence of sealed broods [14,33,39,93], the low efficacies of 50% and 41.8% can be attributed to a high amount of brood in [67] and [73]. Overall, the resulting low median of 50% should not be overestimated.

Over the years, no tendency towards resistance development or loss of efficacy below 70% was observed for trickling oxalic acid, irrespective of dose group.

To rule out resistance masked by dose adaptation, the dose variation over the years must be evaluated as described above. Most of the time, in the “trickling” category, no decrease in the median efficacy immediately before dose increase was observed. Only the period from 2006 to 2008 should be investigated more closely. In 2006, an annual average dose of 33.1 mg/dm^2^ (T_4_) was used, followed by 14.3 mg/dm^2^ (T_2_) in 2007 and 37.3 mg/dm^2^ (T_4_) in 2008. The corresponding annual average efficacies of 85.8% in 2006, 82% in 2007, and 75.6% in 2008 decreased over time, although the dose increased from 2007 to 2008. The efficacy in 2008 was lower than in 2006, even if the average dose was higher in 2006 than in 2008. All of the colonies used in 2008 had broods while testing, and so a lower efficacy despite a higher dose is not surprising, as the colonies from 2006, which led to a higher efficacy value, had no brood at all. A lower efficacy in brood-positive colonies has to be expected in general. After 2008, the median efficacy levels of the dose group T_2_ and T_4_ rose again and resulted in efficient treatment of between 74% and 99%. Additionally, most of the efficacy values in these dose groups were higher than 70% and even higher than 90%. Therefore, the situation described above was not due to resistance development, but from different brood status.

During the rest of the observed period, the average dose ranges were relatively stable between T_2_ and T_4_ combined with a mostly stable efficacy level above 70%. The highest dose group, T_6_, was only used in six years starting in 2004 with a gap between 2013 and 2019, and then used again in 2020. According to the rise in the annual average dose in 2020 from 19.7 mg/dm^2^ (T_2_) to 45.2 mg/dm^2^ (T_5_), the efficacy at this point rose as well. Therefore, this is not an indication of resistance development, because the efficacy did not remain at the same level and correlated positively with the dose increase. Finally, there was no evidence of masked resistance in this category.

### 4.3. Spraying

Over the whole period with available data from 1994 to 2013, the annual median efficacy of spraying oxalic acid was above 70%. Only three of the nine documented median values were lower than 90%, as recommended by the EMA. This fact strongly supports the conclusion of persisting efficacy from spraying oxalic acid.

Taking a closer look at the dose modifications over the years, there was no significant dose modification. The dose ranges were stable around S_2_ from the upper third of S_1_ to the lower third of S_3_. The only great dose adaptation led by an increase in efficacy was found from 1999 to 2003. The dose increased from 14.5 mg/dm^2^ (S_2_) in 1999 to 22.6 mg/dm^2^ (S_3_) after an efficacy drop from 1995 at 96.7% to 1999 at 83.5%. This efficacy decrease can be explained by the brood status, as the colonies in 1995 were broodless, while the colonies in 1999 had broods. Although the average dose in 1995 at 8.3 mg/dm^2^ was lower than in 1999, the efficacy dropped because of the different brood status and lower efficacy in the presence of broods. Then, in 2002 and 2003, the average dose rose and the efficacy also increased, even higher than in 1995. The colonies in 2002 and 2003 had no broods and so the efficacy increases were positively correlated with the dose escalation. Because the efficacy did not remain the same or decrease as the dose increased, there was no evidence of masked resistance in this category.

### 4.4. Exchange via Direct Contact

The individual efficacy values for the category “exchange via direct contact” led to an annual median efficacy of 70% or more, except for three years. In 2005, 2006, and 2022, the median efficacy was 34%, 18.7%, and 69.1%, respectively. In 2005, only one efficacy value of 34% was reported in a literature source [68]. The study was conducted under field conditions with a brood area of 670 square centimetres in September in Canada [68]. The control treatment used for the efficacy calculation was the coumaphos-containing CheckMite+^®^ and the fallen mites were monitored via sticky bottom boards [68]. In the study,, 47.6 milligrams of oxalic acid dihydrate per litre of hive volume was used [68]. Other comparable doses from dose group E_1_ resulted in a median annual efficacy of more than 70%, except for the median in 2011. The median efficacy of group E_1_ in 2011 was obtained in Ref. [67] with a single efficacy value of 48%. The dose in this study was exactly the same as in [68]. In both studies, oxalic acid dihydrate was applied via a vermiculite block soaked in an oxalic acid–ethanol solution [67,68]. None of the other studies included in this category used an ethanol solution. Ethanol is a highly volatile substance. This may explain the lower efficacy due to the short time of effect on *Varroa destructor*. The following year, 2006, had an even lower median efficacy with only one value. The tested hives had no brood and were treated in autumn with a follow-up treatment of Perizin^®^ plus trickling oxalic acid [45]. The dose of 2.6 g of oxalic acid dihydrate per colony was relatively low, similar to the studies mentioned above [45,67,68]. Oxalic acid dihydrate was applied via cellulose strips soaked in an aqueous solution, twice, at 14-day intervals [45]. According to Marinelli et al., the low efficacy was due to the dry conditions in the hive, which inhibited the hydration of the oxalic acid crystals attached to the cellulose strips [45]. Therefore, an effective level of active ingredient could not be achieved. Accordingly, the resistance of *Varroa destructor* is not the reason for the low efficacy. For 2022, the brood status of the study was not reported [37]. The two methods of application resulting in the reported efficacy values of 94.5% and 43.6% differed significantly [37]. Application using a 10% oxalic acid paraffin oil in an undefined “fumigation machine” resulted in lower efficacy [37], whereas 94.5% efficacy was achieved via cellulose strips soaked in an oxalic acid–glycerine–water solution [37]. Four strips per colony corresponded to a volume dose of 440.5 milligrams of oxalic acid dihydrate per litre of hive volume [37]. All of the median annual efficacies of this dose group, E_2_, were greater than 90%. Furthermore, glycerine is a highly viscous and hygroscopic fluid. This characteristic may help the acid to spread among the bees over a longer period of time [22] and lead to a more uniform distribution rate than for other, non-glycerol strip formulations, thereby increasing its efficacy. In contrast, paraffin without any hydroxyl group is less hygroscopic; therefore, oxalic acid does not dissolve in this liquid. It is therefore less well distributed in the hive and, thus, the concentration of the active ingredient, oxalic acid, in the colony is lower and uneven over time. Consequently, the mites are not affected by oxalic acid and, accordingly, the efficacy is lower. These circumstances suggest that the dosage and the chemical property of glycerine are the decisive parameters and that the possible resistance of *Varroa destructor* is not the reason for the unsatisfactory efficacy of an application via paraffin oil.

The annual average dose in this category remained stable around the lower quarter of E_1_ from 2004 to 2016, rising in 2018 to the upper quarter of E_1_ and levelling off in E_2_ after that. From 2018, a new formulation with glycerine was used. The dose increased and the duration of application also increased. The glycerine formulation enabled the application of one device over weeks and still created an effective level of oxalic acid in the hive. Thus, even if the average dose rose from 119.5 mg/L in 2016 to 398.3 mg/L in 2018, the efficacy decreased, because the new formulation with glycerine was only left in the hive for one week in 2018 [83]. In 2019, the efficacy increased to 92.5% because the device was left for four weeks and the dose rose to 512.7 mg/L [37]. Thus, the lower efficacy in 2018, despite the higher dose, resulted from the short application time and failure to reach the effective level of active substance in the hive. Although the annual average in 2018 reached the dose group E_2_, there were still some trials using doses lower than or equal to 400 mg/L. The annual median efficacy in this dose group (E_1_) after 2018 was still higher than 70% and sometimes even higher than 90%. This indicates that the dose escalation in 2018 is not evidence of efficacy loss, but only a modification in the application method, which could be more practicable for the apiarist.

### 4.5. Vaporisation

The annual median efficacy reached at least 70% from 2001 to 2022, except for 2017 and 2021, with 41.4% and 58.7%, respectively.

The median in 2017 derived from three values from one study using different doses that correlated positively with the efficacy values (30%, 41.4%, 69.3%) [55]. All colonies had about six frames of brood during the study [55]. High amounts of brood are generally associated with the reduced efficacy of oxalic acid, which was also observed in the “vaporisation” category, where three (12.3%) values from brood-positive colonies reached an ineffective level of lower than 70% compared to only one (4%) ineffective value from colonies without broods. Thus, the brood status may explain the observed low efficacies. Oxalic acid was applied via the commercial vaporiser VARROX^®^ [55]. No critical test was used, but the mites were counted by sampling with sticky bottom boards and the alcohol washing method [55]. The doses from dose groups V_1_, V_2_, and V_3_, comparable to those used in this experiment, always resulted in a median annual efficacy higher than 70% and, in most cases, even higher than 90%. This fact, combined with the positive brood status, suggests that the low efficacy values in 2017 are not related to resistance development. 

The median efficacy value for 2021 results from one value from one literature source. The five hives used were broodless, and mite monitoring was carried out using the bottom board and powdering method [76]. Two grams of oxalic acid dihydrate was vaporised by the commercial vaporiser Sublimox^®^ [76]. Kolics et al. neither counted mites before treatment nor used a critical test as recommended by the EMA [7]. The efficacy of oxalic acid was estimated by comparing the mite fall after oxalic acid treatment with the mite fall over the whole treatment period including a second treatment using lithium chloride [76]. The mite fall was counted over a period of 8 days after treatment with oxalic acid and 11 days after treatment with lithium chloride [76]. A mite fall over three weeks was described for oxalic acid trickling [99]. Assuming a similar period for vaporisation, mite monitoring over only eight days is far too short; therefore, the efficacy of oxalic acid at 58.7% was probably underestimated. Due to these circumstances, the relevance of this efficacy value is limited for the question of resistance development.

The most commonly used doses in “vaporisation” belonged to groups V_1_ and V_2_. Except for 2011 and 2012, the annual average dose varied between the middle of V_1_ and the middle of V_2_. In 2011, the dose rose from 48 mg/L (V_2_) in 2009 to 97 mg/L (V_3_). The efficacy remained the same (95% and 95.1%). Assuming that broodless colonies were treated more effectively, this should be expected, as the colonies in 2011 had broods, but the ones in 2009 were broodless during the trial. Figure 7 supports this idea: in 2012, the average dose was group V_3_ at 73 mg/L and the efficacy was similar to 2011 at 92.3%. These colonies also had broods. But then, in 2013, the dose decreased to 20 mg/L (V_1_) and the efficacy remained at 91.4%. The difference here is that the colonies were broodless, so a lower dose was still effective. In 2017, only V_2_ was used in brood-positive colonies; therefore, the efficacy decreased significantly to 41.4%. The dose group used in 2022 was still V_2_, but this time without broods. The efficacy rose to 85.6% again. Finally, the annual median of V_2_ was—except for 2011—always higher than 80% or even higher than 90%.

Overall, there was no indication of resistance development in this category.

## 5. Conclusions

Overall, there is no robust evidence in the evaluated relevant literature that *Varroa destructor* has developed resistance to oxalic acid over the past 30 years. Irrespective of the method of application, the efficacy is, in most cases, higher than 70% or even 90%, and outliers can be explained by the different study settings. Due to the small number of efficacy values and their large spread, medians were calculated. While they do not give any indication of resistance against oxalic acid, there is no robust evidence to rule out resistance development. Further research and standardised tests are required to establish continuous monitoring, because the different conditions and study settings clearly limit the validity and comparability of the values.

## Figures and Tables

**Table 1:** Groups of oxalic acid dihydrate areal doses in the “trickling” category.

Group	Areal Dosage Range (mg/dm^2^)	Number and Share of Efficacy Values	Efficacy			References
			Range (%)	Fraction ≥ 70%	Fraction ≥ 90%	
T_1_	0–10	20 (10.8%)	22.2–97.8	11	5	[61,68,96]
T_2_	>10–20	73 (39.5%)	27.9–100	65	49	[14,33,42,52,58,60,61,71,84,85,87,89,96]
T_3_	>20–30	27 (14.6%)	66.1–99.8	26	20	[13,15,18,46,51,54,60,61,64,66,69,81,96]
T_4_	>30–40	19 (10.2%)	5–92.8	12	2	[1,16,32,56,66,73,77,80,91,94]
T_5_	>40–50	6 (3.2%)	72.2–95	6	2	[56,62,69,80,91]
T_6_	>50	7 (3.8%)	8.3–93.7	5	3	[56,67,80,91,92]
T_un_	Unknown; not calculable; another unit	33 (17.8%)	14.5–98.6	24	10	[19,41,43,44,45,50,53,59,63,70,71,72,74,75,85,93,95,97]

**Table 2:** Groups of oxalic acid dihydrate doses in the “spraying” category.

Group	Areal Dosage Range (mg/dm^2^)	Number and Share of Efficacy Values	Efficacy			References
			Range (%)	Fraction ≥ 70%	Fraction ≥ 90%	
S_1_	0–10	9 (31.0%)	67.3–98.3	8	7	[34,35,36,61]
S_2_	>10–20	11 (37.9%)	73–99.42	11	9	[34,35,61,86]
S_3_	>20–30	5 (17.2%)	87.4–99	5	3	[15,34,49,61]
S_4_	>30–40	1 (3.4%)	98	0	1	[34]
S_un_	Unknown; not calculable; another unit	3 (10.3%)	50–95	2	2	[57,90]

**Table 3:** Groups of oxalic acid dihydrate volume doses from “exchange via direct contact” category.

Group	Volume Dosage Range (mg/L)	Number and Share of Efficacy Values	Effectiveness			References
			Range (%)	Fraction ≥ 70%	Fraction ≥ 90%	
E_1_	0–400	6 (33.3%)	34–78.7	3	0	[37,67,68,82,83]
E_2_	>400–900	6 (33.3%)	90.4–94.5	6	6	[37]
E_un_	Unknown; not calculable; another unit	6 (33.3%)	18.7–94	4	3	[22,37,45,47]

**Table 4:** Groups of oxalic acid dihydrate volume doses from “vaporisation” category.

Group	Volume Dosage Range (mg/L)	Number and Share of Efficacy Values	Effectiveness			References
			Range (%)	Fraction ≥ 70%	Fraction ≥ 90%	
V_1_	0–30	5 (20%)	30–94.2	4	2	[17,55,61]
V_2_	>30–60	8 (32%)	41.4–99.6	7	4	[17,37,48,55,61,65,66]
V_3_	>60–100	3 (12%)	69.3–95.1	2	2	[55,66]
V_un_	Unknown; not calculable; another unit	9 (36%)	58.7–96	8	7	[59,63,76,98]

## Data Availability

Newly generated data are described in the article. They were generated out of already existing data from different literature sources shown in the “References” section.
